# Small Doses Which Are Effectual

**Published:** 1886-10

**Authors:** 


### SMALL DOSES WHICH ARE EFFECTUAL.

UNDER this heading a writer in the *Nashville Journal of Medicine and Surgery*, says:

"The tendency in prescribing is to elegance and pleasantness. Although we have capsules, wafers, sugar and chocolate coatings, yet the drug may prove inert by the insolubility of the coating. Since the discovery of various alkaloids, small doses have become more common. If drugs are effectual in small doses frequently repeated, why not prescribe small doses?

But do not understand me to say that we can prescribe for all diseases in this manner. There are some troubles which are only overcome by heroic doses.

In diphtheria, scarlatina, follicular tonsillitis, potassium chlorate in one grain doses every half-hour, affords much relief, and is curative.

One-grain doses of croton chloral every half-hour, in many forms of neuralgia, is beneficial.

In obstinate urticaria, salicylate of soda in two grain doses every half-hour, acts well; also drop doses of balsam of copaiba every half-hour.

The vomiting of drunkards is often helped by half-drop doses of Fowler's solution.

For voting of infants, one grain of calomel to one ounce of lime water; to this add one pint of pure water, and give a teaspoonful of the mixture every ten minutes.

In wheezing and cough of children with bronchitis, good results may be obtained with tartar emetic, one grain to two pints of water; teaspoonful every half-hour.

Sick headache is often relieved by one drop of tinc. nux. vomica every five minutes.

One of our best remedies for inflammation of the bladder is tinc. cantharides, one drop every hour.

In excessive menstruation, fl. ext. ergot has been successfully used in minim doses every half-hour, for six or eight hours before the expected flow. A simple febrile movement, with hot, dry skin, full and bounding pulse, may be relieved by half-drop of tinc. aconite root every half-hour; also useful in acute nasal catarrh.

Sub-acute nasal catarrh, with abundant secretions, is often delayed by minim doses of tinc. belladonna, every half-hour until eight or ten minims are taken.

In malarial fever, when quinine fails, picric acid, gr. 1-8, in combination with ammonia, is used with benefit; also beneficial in pertussis.

In asthma, with indigestion and anæmia, Fowler's solution one drop doses often proves remarkably beneficial.

Apomorphia, gr. 1-20 three or four times a day, often produces brilliant results in spasmodic cough.

